# Effects of Caregiver Interventions for Informal Caregivers of Older Adults With Cognitive Decline

**DOI:** 10.1093/geroni/igab046.2442

**Published:** 2021-12-17

**Authors:** Machiko Tomita

**Affiliations:** University at Buffalo, State University of New York, Buffalo, New York, United States

## Abstract

Objectives: To identify baseline factors and process factors, which indicate changes that are associated with caregiving confidence improvement attributed to caregiver support. Methods: An intervention study using 35 informal caregivers (ICG) of older adults (≥65 years old) with cognitive decline. Recipients of ICGs belonged to the Programs of All Inclusive Care for the Elderly (PACE). Interventions were occupational therapy (OT) support or education about illness and effective caregiving methods, which took place in ICGs’ homes. OT interventions included training to reduce physical strain, and improve time and task organizations, and providing assistive devices). Caregiver confidence was measured using a Visual Analog Scale. Data were divided into two groups: improved confidence and decreased/no-change confidence. Eleven baseline data of care recipients (CRs) and ICGs as well as five process data were analyzed using logistic regression. Results: Baseline factors that differentiated the two groups were ICG’s age, caregiving confidence level, and CR’s cognitive status, of which classification accuracy was 94.3%. Only Zarit Buren Interview (ZBI) score was associated with caregiving confidence change, of which classification accuracy was 74.3%. Younger ICGs, lower cognition, and lower caregiving confidence among baseline factors, and improved ZBI among the process factors were associated with improved confidence. Discussion: Although our interventions prevented 65.7% of caregivers form declining their caregiving confidence, improving caregiving confidence was difficult while CRs’ cognition continued to decline. However, this positive change was possible even CRs had moderate dementia, on average. Personal interventions may be necessary to improve caregiving confidence and reduce ICG’s burden.

